# Adherence to prescription-writing guidelines for outpatients in Southern Gauteng district hospitals

**DOI:** 10.4102/phcfm.v12i1.2263

**Published:** 2020-06-15

**Authors:** Jacques G. Nkera-Gutabara, Laurel B. Ragaven

**Affiliations:** 1 Department of Family Medicine, School of Clinical Medicine, Faculty of Health Sciences, University of the Witwatersrand, Johannesburg, South Africa; 2 Johannesburg Metro Health District, Gauteng Department of Health, Johannesburg, South Africa; 3 Department of Family Medicine, Gauteng Department of Health, Johannesburg, South Africa

**Keywords:** medical prescription writing, prescribers, adherence to guidelines, scoring

## Abstract

**Background:**

Medical prescription writing is legally and professionally regulated in order to prevent errors that can result in patients being harmed. This study assesses prescriber adherence to such regulations in primary care settings.

**Methods:**

A cross-sectional study of 412 prescriptions from four district hospital outpatient departments (OPDs) was conducted in March 2015. Primary outcome data were obtained by scoring prescriptions for accuracy across four categories: completion of essential elements, use of generic names of medications, use of recommended abbreviations and decimals and legibility. Secondary outcome data sought associations between accuracy scores and characteristics of the OPDs that might influence prescriber adherence.

**Results:**

Completion of the essential elements, including patient identifiers, prescriber identifiers, treatment regimen and date scored 44%, 77%, 99% and 99% respectively. Legibility, the use of generic names of medications and the use of recommended abbreviations and decimals scored 90%, 39% and 35%, respectively. Only 38% of prescriptions achieved a global accuracy score (GAS) of between 80% and 100%. A significant association was found between lower GAS and the number of prescriptions written per day (*p* = 0.001) as well as with the number of prescribers working on that day (*p* = 0.005), suggesting a negative impact on prescribers’ performance because of workload pressures.

**Conclusion:**

Low GAS values indicate poor adherence to prescription-writing regulations. Elements requiring substantial improvement include completion of patient and prescriber identifiers, use of generic medication names and the use of recommended abbreviations and decimals. This study provides baseline data for future initiatives for improvement in prescription-writing quality.

## Introduction

Medical prescriptions are often the last encounters patients have with healthcare practitioners. Their writing is regulated by professional guidelines and the law.^1,2,3,4^ Prescription-related inaccuracies may impact negatively the quality of care and compromise patient safety, and they may lead to medication errors that have significant social, financial and legal consequences.^5,6,7,8^ Such errors can arise from errors related to medication prescribing, dispensing, administration or patient compliance;^9,10^ however, the origin of majority of errors noted in the medical prescription can be traced to writing by healthcare professionals,^11^ which can then have knock-on effects such as delayed dispensing.^12^ Prescription-writing errors account for up to 70% of medication errors that could potentially result in adverse effects.^13^

### Prescription-writing standards

There have been international and domestic attempts at standard setting to determine the essential elements to be included when writing a prescription for medication. In South Africa, we are guided by the World Health Organization’s (WHO) ‘Guide to Good Prescribing’,^1^ the National Department of Health’s ‘prescription writing’ guidelines^2^ and, since 2017, the ‘General Regulations’ of the *Medicines and Related Substances Act* 101 of 1965 (Regulation 33),^4^ which were updated from the 2003 Regulation 28.^3^ While there is a great deal of overlap between these three sets of guidelines, they are however not all the same. The essential elements from these three different sources, which came into effect in 2015, are summarised and compared in Table 1, with the last column showing those that have been selected for this study. The justification for inclusion is explained in the footnotes of Table 1.

**TABLE 1:** Comparative summary of elements essential to a medical prescription, according to international and domestic best practice.

Medical prescription elements and their components	WHO^1^	EML-2014^2^	*Medicines and Related Substances Act* 101 of 1965 (Regulation 28)^3^	Included for audit in this study
**Elements and components**				
Date of prescription	+	+	+	+
**Patient identifiers**				
Names	+	+	+	+
File number		+		+
Address†	+	+	+	
Age	+	+	+	+
Sex			+	+
Weight††		+		+
**Treatment regimen**				
Medication name in full (either trade or generic)†††	+	+	+	+
Strength	+	+	+	+
Dosage	+	+	+	+
Frequency or interval	+	+	+	+
Duration		+	+	+
Quantity§	+		+	
Repeats		+	+	+
Use of medication generic name in full†††	+	+	+	+
Use of abbreviations and decimal points§§	+	+		+
**Prescriber identifiers**				
Name	+	+	+	+
Address†	+		+	
Qualification or degree			+	+
Professional registration number†			+	+
Contact number†	+	+		
Signature	+	+	+	+
Legibility of handwriting		+	+	+
Permanent copy¶)			+	
Diagnosis¶¶			+ (with patient’s consent)	
Warnings#	+			

OPD, outpatient department; WHO, World Health Organization; HPCSA, Health Professions Council of South Africa; MP, Medical Practitioner; EML, Essential Medicines List.

† , The patient and prescriber addresses and the prescriber contact number, although legally required, were not included for assessment in this study, as all prescriptions were dispensed internally via the hospital OPD where both the patient and prescriber were situated. No outside prescriptions were considered. Similarly, practice number was replaced by the prescriber’s professional registration number (e.g. HPCSA MP number) because all prescribers worked in the public sector.

†† , The patient’s weight is mandatory for children, and it is also required for the frail elderly and where dosing is weight dependant; this allows for a double check by the dispenser.

††† , The medication name is scrutinised for full name use (not abbreviated) and separately for generic name use.

§ , Quantity of medication was left out as the dosage, frequency and treatment duration are its equivalent, and in most settings, the calculation of quantity of medication to dispense is a function of the pharmacy.

§§ , Importantly, there are no legally approved abbreviations for medications. The WHO Guide to Good Prescribing^1^ and the Essential Medicines list^2^ caution against the use of possibly confusing abbreviations because of the risk of misinterpretation. Similarly, decimal points should be employed only where they are unavoidable.

¶ , A permanent copy of the prescription is kept by the pharmacy.

¶¶ , The diagnosis is found in the patients notes to which the prescription is appended.

# , Warnings are delivered verbally by pharmacist at the time of dispensing.

Despite the existence of these professional norms and legal standards, significant challenges related to adherence by prescribers are reported in international literature.^11,12,13^

Research on the reasons for poor quality of prescription writing deals with it from various perspectives, including the setting in which care is provided (from outpatient primary care to hospital wards and to specialised intensive care units) as well as the professional profiles of prescribers (amongst students in different professional programmes to consultants and university faculty).^14,15,16,17^ While generalising, there seems to be no single factor accounting for adherence or non-adherence to prescription-writing guidelines.

### Prescription-writing errors

Indicators used to assess the accuracy of a medical prescription in compliance with writing regulations include the completion of essential elements, the use of generic names for medication, the use of recommended abbreviations and decimal points and, finally, script legibility if handwritten. The essential elements that must be completed are further broken down into ‘the date of prescription’, ‘the patient identifiers’, ‘the treatment regimen’ and ‘the prescriber identifiers and signature’.

Errors of omission or incompleteness in prescription writing are the most reported ones.^14,15,16,17,18,19,20,21,22,23,24,25,26,27,28^ Other errors include the use of trade names of medications rather than their generic equivalents and the use of non-recommended abbreviations and decimal points^18,29,30^ Handwritten prescriptions are consistently found to be more prone to errors than electronic ones.^18^ Junior doctors are reported to make more errors when prescribing medication, with insufficient knowledge and the work environment which includes heavy workload, time pressures and the influence of the prescribing habits of the seniors cited as contributing factors.^19,20^ Illegible handwritten scripts are also reported as a frequent cause of error.^28,31,32^ Legibility has been assessed using different methods including optical computer software and subjective scales, with the legibility of letter words (such as drug names) being worse than that of numbers (such as frequency of dosage).^33,34,35^ A validated tool, the Prescription Quality Index (PQI), provides indicators to measure legibility in chronic diseases using a three-point Likert scale, with scores of 0 for ‘illegible’, 1 for ‘barely legible’ and 2 for ‘legible’.^36^

### Assessing and reporting on the quality of prescription writing

The literature assesses and reports on the quality of prescription writing in various ways, including simple counts of present or missing elements of the prescription, graded scoring for the completion of elements (not completed, partially completed or fully completed) and classification of acceptability into categories according to scores realised.^36,37,38,39^ Scaling and weighting have also been used in assessing the quality of a prescription; however, this is not required, nor is validation, for components that are purely descriptive in nature.^29,30,36,37,38,39,40,41^ Various aspects of quality assessment and reporting have been incorporated into this study.

### Prescription-writing quality improvement

Improvement in the quality of medical prescription writing is achievable. Experience from other settings indicates, for example, that prescription audits in conjunction with education on rational pharmacotherapy improved the prescribing skills of fourth-year medical students.^42^ Serial audits and targeted interventions including educational strategies (such as feedback of audit results for prescribers on prescribing and medication errors) and changes in systems of practice (such as modifications to medication charts, publication of hospital-wide prescribing standards, an alert notification system and the implementation of electronic health records) have resulted in improvements for all of the indicators of medical prescription quality standards.^21,43^ A modified out-patient prescription chart with prompts for all the required elements to reduce prescription errors has been recommended,^22^ as were, in other studies, ongoing education programmes, follow-up reminders, regular audits and feedback sessions and the use of prescribers’ self-inking stamps.^16,17^ A sevenfold decrease in prescription errors was reported with the introduction and adoption of electronic prescribing,^44^ which is consistent with 2008 systematic reviews on this subject.^45,46^

To our knowledge, there is no published study on adherence to South African regulations for medical prescription writing in primary care settings. An unpublished 2014 in-house audit of prescription writing in the outpatient department (OPD) of one district hospital in Southern Gauteng raised concerns, providing the impetus for this study, when it was found that 83% of 400 prescriptions had only achieved less than a 40% global accuracy score (GAS), with the remaining 17% of prescriptions scoring between 40% and 80%, and 0% scoring between 80% and 100% in compliance with prescription-writing regulations.^47^

The purpose of this study therefore was to assess the adherence of prescribers across Southern Gauteng district hospitals to prescription-writing regulations and examine potential barriers in order to raise awareness and provide baseline data for future prescription-writing quality improvement initiatives.

## Methods

### Study design

This is a cross-sectional study dealing with the accuracy in prescription writing with regard to adherence to regulations amongst all levels of prescribers (medical students, primary healthcare nurses, clinical associates, medical interns, medical officers and medical specialists) working in a primary care setting who were blinded to the study.

### Study site

The study was conducted in the OPDs of four district hospitals in Southern Gauteng, representing the University of the Witwatersrand Family Medicine training platform.

### Study population

The study included all medical prescriptions issued at the OPDs in Southern Gauteng district hospitals in March 2015, the day prior to the researcher’s site visit.

### Sample size

Considering the results of previous research that indicated the range of 1.3% – 85.9% for accurately written prescriptions, with a precision of 5% and 95% confidence level at a power of 80%, using Stata12^®^ (Stata Corp, College Station, TX, USA), the calculated sample size was 374 prescriptions.

### Sampling

The selection of the hospitals to be surveyed was randomised as follows: one hospital was randomly selected from each of the three health districts with more than one district hospital; the fourth district hospital was in a district with only one such hospital. The names of the hospitals were written on A4 size papers that were folded four times, placed in a bucket and a researcher’s colleague would pick one from the bucket for districts where there was more than one hospital. The order in which the hospitals were visited was from the furthest to those closest to the Wits University Medical School. A convenient sampling of a minimum of 100 consecutive OPD prescriptions written for adult and child patients and dispensed the previous day was assessed per hospital to ensure reaching the calculated sample size.

### Data collection instrument

Nineteen essential elements of prescription writing were selected from existing regulations for this study (Table 1). These were compiled into a single data collection tool (Appendix 1), which was a Microsoft Excel™ spreadsheet, with variable codes and scoring. All data were manually extracted by the researcher (J.G.N.-G.) through a review of real prescriptions accessed from the pharmacies the day after all medications had been dispensed and kept in boxes by the pharmacist for this purpose.

### Variables

For each prescription, the completion of the essential elements, the use of the generic names of medications, the use of recommended abbreviations and decimal points, and prescription legibility were evaluated, and an accuracy score out of 38 total points was determined. Each element was assessed, with scoring assigned as 0, 1 or 2 for ‘not completed’, ‘partially completed’ or ‘fully completed’, respectively. The use of generic names of medications, recommended abbreviations and decimal points were scored 0, 1 or 2 for ‘not used’, ‘partially used’ or ‘fully used’, respectively. The medicine name was scored twice, but for different aspects: firstly, for full and correct writing (not abbreviated) with either the trade or generic name in the treatment regimen; and, additionally, for the use of its recommended generic form.

Legibility was scored according to the PQI^35^ as 0, 1, or 2 for ‘illegible’, ‘barely legible’ or ‘legible’, respectively, as subjectively determined by the researcher.

Prescriptions for children were particularly checked for the completion of details regarding age and weight. Prescriptions for controlled substances were checked for full letter writing of dosages and quantities. In the South African context, this refers particularly to schedules 5 and 6 substances^1^ which would be available in primary care facilities, and necessitate both medical diagnosis and management, and also enhanced control of supply.^48^

For prescriptions requiring two signatures (such as that of a medical student to be countersigned by a medical officer), the medical officer’s signature will be used to determine the prescriber, unless there is no counter-signature, which would constitute a procedural omission. Prescriptions by community service doctors and registrars were counted as those given by medical officers as they could not be distinguished. Finally, other characteristics for each of the district hospital’s OPD included the average number of OPD prescriptions per day and the number and professional categories of prescribers present in OPD on the day the prescriptions were written.

### Global accuracy score

A GAS for each prescription was determined by calculating the total percentage achieved out of 38 possible points for the 19 prescription elements considered (Table 1). Each GAS was then classified into one of the following four scores: 100%, 80% – 100%, 40% – 79% and less than 40%. The desired prescription-writing accuracy score, or gold standard, is 100%; however, based on previous earlier findings,^47^ its expected rate was negligible.

Accuracy score category ranges of 80% – 100%, 40% – 79% and less than 40% were therefore used. These score ranges were purely descriptive, and are similar to the score ranges employed by the Council for Health Service Accreditation of Southern Africa (COHSASA)^49^ which is a private non-profit entity focussing on quality improvement and conducting healthcare facility accreditation.

## Outcome measures

### Primary measures

The completeness of essential elements of a prescription, the use of the generic names of medications, the use of abbreviations and decimal points, the legibility of the prescription, and a GAS for each prescription were determined.

### Secondary measures

Calculated associations between the GAS categories with:

- the average number of OPD prescriptions per day
- the number of OPD prescribers on that day
- the prescribers’ professional categories, grouped either as allied health professionals: categories 1–3, or medical doctors: categories 4–6
- the type of prescription (adult, child and controlled substance).

### Data analysis

After the electronic and direct entry onto the specially designed data collection tool in MS Excel, the data were cleaned, sorted and then exported into Stata 12^®^ software for analysis. Descriptive analysis of the categorical data was conducted and results were presented in tables as frequencies and percentages as well as in figures. For elements with multiple components, the mean score and standard deviation (s.d.) were also determined. Cross-tabulation was used to explore possible associations between primary and secondary outcomes using statistical tests including the student’s *T-*test, Pearson’s Chi-squared test, Fisher’s exact test and Kuskral–Wallis test. A *p*-value of < 0.05 was considered statistically significant. Logistic regression analysis was conducted where associations were found.

### Ethical consideration

The approval to conduct this study was granted by the Human Research Ethics Committee (Medical) of the University of the Witwatersrand (clearance certificate number M141179). The CEOs of the four hospital issued authorisation letters granting permission to access their facilities for purposes of the study. A further undertaking form was signed with each hospital records administrator to not record and to preserve at all times the anonymity of patients and prescribers.

## Results

A total of 412 prescriptions were analysed across all districts, with each of the hospital sites contributing approximately 25% of the entire sample. Table 2 is a descriptive account of the specific characteristics for each district hospital OPD as well as for the four district hospitals combined. Significant differences were found between the hospitals with regards to the average number of prescriptions per day, the number of prescribers on the day and the prescribers’ professional category mix. The average number of prescriptions per day varied between 100 and over 300, and that of prescribers varied between six and eight on the days studied. Medical officers constituted 83.25% of the prescribers overall. Adult patients accounted for 96.12% of all prescriptions written.

**TABLE 2:** Outpatient department site-specific characteristics per district hospital and for all of Southern Gauteng.

Variable	Health district or district hospital sampled										*p*
	COJ or South Rand		West Rand or Yusuf Dadoo		Ekurhuleni or Bertha Gxowa		Sedibeng or Kopanong		Southern Gauteng total		
	*n* (101)	% (24.51)	*n* (105)	% (25.49)	*n* (105)	% (25.49)	*n* (101)	% (24.51)	*n* (412)	% (100)
**Sites-specific characteristics**											
Average number of OPD prescriptions per day†	300+	-	100–200	-	201–300	-	201–300	-	-	-	0.001
Number of OPD prescribers present on the day††	7	-	8	-	6	-	8	-	-	-	0.001
**Type of prescriptions**											0.2
Adults	96	95.04	102	97.14	100	95.23	98	97.02	396	96.12	
Paediatrics	4	3.96	1	0.95	4	3.96	0	0.00	9	2.18	
Controlled substances	1	0.99	2	1.90	1	0.95	3	2.97	7	1.70	
**Prescriber categories**									-	-	0.001
1. PHC nurses	0	00	6	5.57	0	00	7	6.93	13	3.16	
2. Medical students	0	00	1	0.95	0	00	0	00	1	0.24	
3. Clinical associates	0	00	7	6.66	4	3.80	5	4.76	16	3.88	
4. Medical interns	19	18.81	4	3.80	0	00	10	9.52	33	8.01	
5. Medical officers	81	80.19	87	82.85	101	96.19	74	73.26	343	83.25	
6. Specialists	1	0.99	0	00	0	00	5	4.95	6	1.46	

OPD, outpatient department; COJ, City of Johannesburg; PHC, Primary Health Care.

† , Data for average number of OPD prescriptions were sourced from Pharmacy statistics.

†† , Data for number of OPD prescribers present on the day were sourced from OPD operational managers.

**FIGURE 1:**
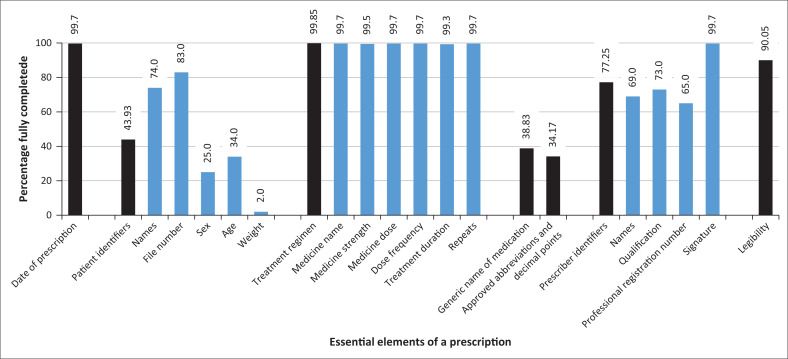
Scores for prescription completeness and accuracy across 19 essential elements for Southern Gauteng district hospitals (*N* = 412).

Figure 1 illustrates the full completion in percentages of a prescription’s 19 essential elements across the 412 prescriptions assessed in Gauteng South; the raw count and percentages showing fully completed, partially completed and not completed elements of the prescription for Gauteng South and for each of the four district hospitals are included as Appendix 3. It is worth noting that one prescription was signed for by a student and not counter-signed by a medical officer, and existing internal memos allow clinical associates and medical interns to write OPD prescriptions, notwithstanding the challenge with regard to the legal status of clinical associates as authorised prescribers, as this category has not been accommodated in the schedules as required by the *Medicines and Related Substances Act*.

Prescribers achieved rates of 90% and over for completion of the date of the prescription, the treatment regimen, the prescriber’s signature and handwriting legibility. The patient’s file number was indicated in 83% of prescriptions. Lower rates were seen for the use of generic names of medications (39%), abbreviations and decimal points (34%) and for other components of patients and prescribers’ identifiers. Particularly, low completion rates were seen for patients’ sex (25%), age (34%) and weight (2%).

Table 3 is a compilation of GASs: 37.52% of prescriptions scored 80% – 100%, whilst 62.38% scored 40% – 79%. The mean score out of 38 was 27.92 (73.38%) ± 5.53 s.d.

**TABLE 3:** Global accuracy scores per prescription and by elements.

Variable	Health district or district hospital										*p*
	Southern Gauteng		COJ orSouth Rand		West Rand orYusuf Dadoo		Ekurhuleni orBertha Gxowa		Sedibeng orKopanong		
	*n* (412)	%	*n* (101)	%	*n* (105)	%	*n* (105)	%	*n* (101	%	
**Global accuracy of prescriptions written: scores out of 38**											0.001
100%	7	1.69	0	-	0	-	2	1.90	5	4.95	
80% – 100%	155	37.52	16	15.84	33	31.43	62	59.05	44	43.56	
40% – 79%	257	62.38	85	84.16	72	68.57	43	40.95	57	56.44	
0% – 39%	0	-	0	-	0	-	0	-	0	-	
Score out of 38: mean (s.d.)	27.92	5.53	24.62	4.97	25.79	5.39	31.2	4.28	30.03	4.49	
**Date of prescription**											0.9
100%	411	99.75	101	100	105	100	105	100	100	99.09	
80% – 100%	411	99.75	101	100	105	100	105	100	100	99.09	
40% – 79%	0	0	0	-	0	-	0	-	0	-	
0% – 39%	1	0.024	0	-	0	-	0	-	1	0.01	
**Patient identifiers**											0.001
100%	8	1.94	0	-	0	-	2	1.90	6	5.94	
80% – 100%	101	24.51	0	-	0	-	69	65.71	32	31.68	
40% – 79%	200	48.54	27	26.73	84	80.00	22	20.95	67	66.34	
0% – 39%	111	26.94	74	73.27	21	20.00	14	13.33	2	1.98	
Score out of 10: mean (s.d.)	4.39	2.72	2.25	2.07	3.54	1.73	6.27	2.81	5.47	2.15	
**Treatment regimen**											0.9
100%	410	99.51	101	100	104	99.04	104	99.04	101	100	
80% – 100%	410	99.51	101	100	104	99.05	104	99.05	101	100	
40% – 79%	2	0.49	0	-	1	0.95	1	0.95	0	-	
0% – 39%	0	-	0	-	0		0	-	0	-	
Score out of 12: mean (s.d.)	11.98	0.24	12	0.00	11.96	0.39	11.97	0.29	12	0.00
**Use of generic names of medications**											0.3
100%	160	38.83	33	32.67	42	40	40	38.09	45	44.55	
80% – 100%	160	38.83	33	32.67	42	40	40	38.09	45	44.55	
40% – 79%	0	0	0	-	0	-	0	-	0	-	
0% – 39%	252	61.16	68	67.32	63	60	65	61.90	56	55.44	
**Use of recommended abbreviations and decimal points**											0.06
100%	143	34.70	30	29.70	40	38.09	29	21.61	44	43.56	
80% – 100%	143	34.70	30	29.70	40	38.09	29	21.61	44	43.56	
40% – 79%	0	-	0	-	0		0	-	0	-	
0% – 39%	269	65.29	71	69.30	65	61.90	76	72.38	57	56.43	
**Prescriber identifiers**											0.001
100%	93	22.57	0	-	0	-	67	63.80	26	25.74	
80% – 100%	241	58.50	36	35.64	40	38.10	88	83.81	77	76.24	
40% – 79%	90	21.84	36	35.64	21	20	17	16.19	16	15.84	
0% – 39%	81	19.66	29	28.71	44	41.90	0	-	8	7.92	
Score out of 8: mean (s.d.)	6.18	2.40	5.17	64.62	4.82	60.25	7.67	95.87	7.06	88.25	
**Legibility**											0.001
100%	371	90.05	96	95.05	95	90.48	103	98.1	77	76.24	
80% – 100%	371	90.05	96	95.05	95	90.48	103	98.1	77	76.24	
40% – 79%	41	9.95	5	4.95	10	9.52	2	1.90	24	23.76	
0% – 39%	0	-	0	-	0	-	0	-	0	-	

OPD, outpatient department; s.d., standard deviation.

The inability to achieve a desired GAS of ≥ 80% was noted for the following elements: patient identifiers (24.51%), the use of a medication’s generic name (38.83%). the use of recommended abbreviations and decimal points (34.70%) and prescriber identifiers (58.50%). High GAS values of ≥ 80% were noted for the following prescription elements: date of prescription (99.75%), treatment regimen (99.51%) and legibility (90.05).

**FIGURE 2:**
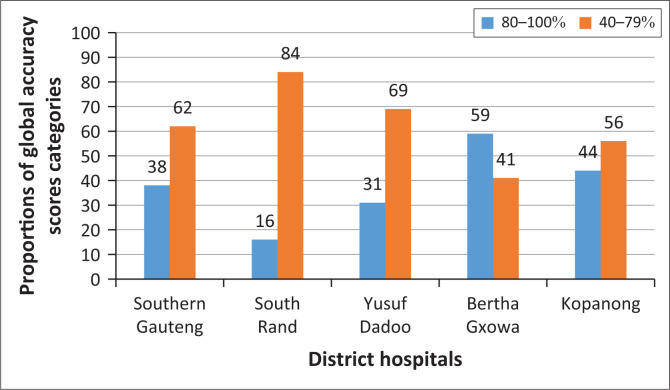
Distribution of global accuracy scores overall and per district hospital: those achieving 80% – 100% versus 40% – 79%.

The distribution of the GAS categories across the district hospitals in Southern Gauteng is indicated in Figure 2. Only in Ekurhuleni did more than half (59%) of the audited prescriptions achieve a score of 80% – 100%.

We examined possible associations between specific characteristics of each OPD and prescription-writing GASs, which are summarised in Table 4. Significant negative associations were found between the ability to achieve a GAS of 80% – 100% and the number of OPD prescriptions per day (*p* = 0.001) and the number of OPD prescribers on the day (*p* = 0.005). No associations were found between the prescription type or prescriber category and adherence to prescription-writing guidelines.

**TABLE 4:** Associations between outpatient department-specific characteristics and prescription-writing global accuracy scores.

OPD characteristic	Accuracy score 80% – 100%		Accuracy score 40% – 79%		Test & *p*-value
	*n* (155)	%	*n* (257)	%	
**Number of OPD prescriptions per day**					
100 – 200	33	31.43	72	68.57	Pearson’s Chi^2^ 0.001
201 – 300	106	51.45	100	48.54	
300+	16	15.84	85	84.16	
**Number of OPD prescribers on the day: mean (s.d.)**	7.10	0.08	7.33	0.05	Student’s *T* 0.005
**Prescriber professional category**					Pearson’s Chi^2^ 0.107
Allied health professionals	18	4.37	137	33.25	
Medical doctors	45	10.92	212	51.46	
**Prescription type**					Pearson’s Chi^2^ 0.480
Adult	147	35.68	249	60.44	
Paediatric	4	0.97	9	2.18	
Controlled substances	3	0.73	4	0.97	

OPD, outpatient department; s.d., standard deviation.

Seeking to understand the effects of the number of prescriptions written per day as well as of the variability in the number of providers on GAS values, we conducted logistic regression analysis, which indicated that the number of OPD prescriptions per day and the number of OPD prescribers on the day were significant predictors of GAS values (Table 5).

**TABLE 5:** Logistic regression model for predicting global accuracy scores.

Variable (characteristics)	Odds ratio	95% CI	*p*
**Number of OPD prescriptions per day**			
100 – 200	Ref		
201 – 300	1.68	0.95–2.98	0.073
300+	0.30	0.14–0.62	0.001
Number of OPD prescribers per day	0.73	0.55–0.96	0.027

OPD, outpatient department; CI, confidence interval.

Significantly, the hospital with more than 300 OPD prescriptions per day was 70% less likely to score 80% – 100% compared to the hospital with 100–200 prescriptions per day (odds ratio [OR]: 0.30; 95% CI: 0.14–0.62).

The relationship between the number of OPD prescribers (continuous variable) and GAS indicates that an increase in the number of prescribers on a day was associated with an odds ratio of 0.73 in the likelihood of scoring between 80% and 100% (OR: 0.73). In other words, with every increase in the number of prescribers, there is a reduced likelihood of scoring between 80% and 100%, or reaching the target goal. Although this seems counterintuitive in light of the argument that high workload increases the likelihood of a reduced GAS, it suggests, using advanced statistics beyond the scope of this study, that it is possible to calculate optimal numbers of OPD prescriptions and OPD prescribers on any given day.

## Discussion

To the best of our knowledge, this is the first effort in South Africa to develop a single tool that combines domestic regulatory and international best practice models to audit prescription-writing accuracy in the country’s primary care settings. The results of this study provide a baseline understanding of challenges with prescribers’ adherence to prescription-writing regulations. The results also suggest some possible reasons for omissions and inaccuracies in prescription writing that could guide future quality improvement initiatives.

While sampling was restricted to four district hospitals’ OPDs in Southern Gauteng, the required sample size for data collection was exceeded (374 intended; 412 actual), lending strength to the results. It was also apparent that not all the sites were in fact the same to start with, with statistically significant differences between the sites in relation to average number of OPD prescriptions per day, average number of providers and the professional category of prescribers (Table 2). These differences persisted in terms of GASs across the four sites, with interesting variations.

The composite GAS indicates that only seven (1.69%) of the 412 prescriptions assessed achieved the desired 100% or gold standard, and only 155 prescriptions (37.52%) scored 80% – 100%. In Bertha Gxowa and Kopanong district hospitals, 59% and 44% of the prescriptions achieved the GAS of 80% – 100%,respectively, compared to 16% and 31% in South Rand and Yusuf Dadoo district hospitals, respectively. This is a significant difference (Kruskal–Wallis: *p* = 0.001), largely attributed to the higher scores achieved for completion of patient and prescriber identifiers which, if not counted, would bring all the four district hospitals to similar lower GASs (Kruskal–Wallis: *p* = 0.9).

Out of 38 total possible points, the mean score for all 412 prescriptions was 27.92 (73.38%) ± 5.53 s.d. In Ife, Nigeria, Erhun et al.^14^ reported global percentages of 1.3% and 85.9% of completely filled medical prescriptions, respectively, from a health centre and a teaching hospital, and in France, Fourgon et al.^25^ reported 17% of fully accurate ambulatory patient prescriptions. Similar to our settings, most of these deficiencies pertained to omission errors.

The main omissions found in this study related to patient identifiers and prescriber identifiers in 56% and 23% of prescriptions, respectively. This may be explained by the practice of prescriptions being attached to the patient’s file which already contains all of the patient’s particulars, including the address, telephone contacts and medical diagnostic information. The prescriber in a busy OPD would rarely take the time to re-transcribe these details from the file to the prescription, and it seems this omission also occurs when it comes to write his or her own identifiers.

Practices reported in the literature to enable improved completion of these required identifiers include the consistent use of a sticker or label with the patient’s details (with the birth date rather than the age) and the mandatory acquisition and consistent use of self-inking stamps with the required prescriber identifiers with only a signature required on the prescription.^23,24^ These would be low-cost improvement measures as the sticker or label printing infrastructures already exist in the OPDs and self-inking stamps are not expensive.

High prescriber adherence to regulatory guidelines included completion of the treatment regimen (99%). This is possibly because of the dispensers who, as the last gate keepers, created an institutional culture that places higher emphasis on the treatment regimen writing aspect of the prescription. This is in contrast to other settings where omissions of various components of the treatment regimen have been reported, with some even up to 91% incompleteness.^31^

Similarly, the date of the prescription was present 99% of the time; this was possibly because it is almost always stamped on the prescription chart by the clerk at the time of the patient’s registration. In contrast, the date of prescription was omitted in up to 18% of prescriptions in the reported literature.^26^

Of note in this study is the fact that the generic names of medications were not used for 61% of prescriptions, and non-recommended abbreviations and decimal points were used for 65% of prescriptions. Similarly, high proportions of poor compliance in this regard are reported in the literature.^19,29^ The Human Sciences Research Council in South Africa found in 2005 that amongst general practitioners only 11% prescribed by generic name and generic prescribing was below 50% in public facilities.^50^ Education and individual prescribers’ efforts can improve on this at little cost.^21^

As has been evidenced elsewhere, the adoption of an electronic health record with electronic prescribing that includes standardised fields with prompts for certain patient conditions may result in greatly improved accuracy in all prescription-writing elements considered in this study. This may appear to be a high tech or high cost measure, but may be worth the investment given its positive impact on quality and reducing medication error,^44,45,46^ especially in our environment with increasing malpractice and adverse event litigation.

Significant differences were noted between the district hospitals for GAS values and the essential elements of patient identifiers, prescriber identifiers and legibility. Although no association was found between the prescription type (noting the small numbers of paediatric and controlled substances prescriptions) and the prescriber’s professional category, performance differences have been reported between prescriber categories in the literature.^16,17,18,19^ This also could have happened because not all categories of prescribers were present in the OPD on the day prior to data collection. Therefore, other prescribers at that facility would have been missed and potential differences never examined. Similarly, the significant difference in legibility scores may have been caused by one prescriber in a particular district hospital on that day.

The negative associations found between GASs and the number of OPD prescriptions and prescribers per day are suggestive of a negative impact of a heavy workload on the accuracy in prescription-writing, as also reported by Ajemigbitse et al. in Nigeria.^19^ As suggested by the logistic regression model for predicting GAS values in Table 5, it is possible to determine an optimal number of prescriptions and prescribers per day to minimise inaccuracies, and performance may be even further enhanced if e-prescribing or a computerised physician order entry (CPOE) system could be introduced,^44,45,46^ notwithstanding its high cost, especially given that handwritten prescriptions are consistently found to be more prone to errors than electronic ones.^18^ In limited resource settings, however, prescription-writing quality improvement may still be achieved by using simple low-cost methods such as regular audits and feedback sessions, and the use of prescribers’ self-inking stamps as highlighted above.^16,17^

Several limitations may restrict the generalisability of this study. The lack of diversity in prescription types was discovered only after data collection. It seems that paediatric medical records were kept in separate paediatric clinics within the OPDs and that ‘controlled substances’ records were also usually separated from other records for the purposes of oversight; moreover, as these same categories of patients attended OPD on particular days, their prescriptions may have been missed in the survey. An important additional type of prescription would be that of the elderly, which we did not examine specifically. Importantly, the ‘correctness’ of the medication as it correlated with the medical condition being treated and adherence to treatment guidelines by prescribers in their choice of medication were not assessed. Nor were prescriptions differentiated between new and repeats, or scripts for single versus multiple drugs. Also the snapshot or 1-day survey may have reduced the variability amongst the prescribers; and, to understand the impact of the numbers of patients and prescribers, the sampling mechanism could be refined. Inclusion of these aspects may enhance the results of a future study.

A combination of legal requirements and professional guidelines, drawn from local and international sources, was used in selecting variables in this study (Table 1). The legally binding requirements were drawn from the law in application at the time of data collection^3^ and included amongst the prescriber’s identifiers the ‘practice number’. However, ‘professional registration number’ was used as it was required as a best practice although it was not the legal requirement then. The General Regulations to the *Medicines and Related Substances Act* issued in 2017^50^ replacing the one of 2003^3^ modified the legal requirement to be included in the prescriber’s identifiers from the ‘practice number’ to ‘registration number with the relevant statutory health council’, that is, the ‘professional number’ which identifies the individual prescriber better. The ‘practice number’ is not used for in-house district hospital OPD prescriptions. Its use as a variable might have had some impact on the results obtained, but not on the associations observed.

The 90% legibility score observed in this study is at odds with the literature where much higher proportions of illegible scripts are reported.^16,17,18,19^ Also in the assessment of legibility, all the scoring was done by the same person, the researcher alone. His familiarity with the OPD medicines and the environments could introduce a researcher bias. However, on-site pharmacists anecdotally reported difficulty reading only less than one in 10 scripts.

## Conclusion and recommendations

This study revealed that in Southern Gauteng district hospitals, of 412 OPD medical prescriptions surveyed, one-third achieved a GAS of 80% – 100%, with two-thirds scoring between 40% and 79% for compliance with prescription-writing regulations. The most common deficiencies relate to the omission of patient and prescriber identifiers, with completion rates of 43.93% and 77.25%, respectively. Also, medication generic names and recommended abbreviations and decimal points were used in only 38.83% and 34.17% of prescriptions, respectively. Associations were found between accuracy scores and the number of OPD prescriptions per day (*p* = 0.001) and the number of OPD prescribers on the day (*p* = 0.005), pointing to a negative impact of high patient volumes and multiple providers on reducing the accuracy of prescription writing.

A prescription-writing quality improvement programme would be an appropriate framework to implement recommendations pertaining to institutions or systems and individual prescribers. These recommendations include low-cost or low-tech measures, such as the consistent availing and use of pre-printed stickers with the patient’s particulars on prescriptions and the mandatory use of self-inking stamps with the required particulars for prescribers. Reinforcement is also required regarding the consistent use of generic names of medications and being vigilant about the use of abbreviations and decimal points. E-prescribing, although a high-cost endeavour for our limited resources settings, may be considered given its positive impact elsewhere on the quality of prescription writing and medication error reduction.

## Footnotes

1 Schedule 5 substances include psycho-active medicines such as sedatives and antidepressants (e.g. Benzodiazepines, Tramadol); they have a low to moderate potential for abuse or dependence. Schedule 6 substances are therapeutic narcotics and narcotic painkillers (e.g.Pethidine, Morphine, Tilidine); they have a moderate to high potential for abuse or for dependence.
